# A novel non-destructive testing method for turbine disks using dual array ultrasonic transducer

**DOI:** 10.1038/s41598-022-12622-6

**Published:** 2022-06-08

**Authors:** Lichen Teng, Zhenggan Zhou

**Affiliations:** School of Mechanical Engineering and Automation, Beihang University, Beijing, 100083 China

**Keywords:** Aerospace engineering, Mechanical properties, Metals and alloys, Mechanical engineering

## Abstract

A novel dual array inspection method for detecting the diffusion bonding defects of superalloy turbine disk has been proposed in this study. The influence of relative position between the planar defect and acoustic source has been analysed, and based on which, the transmission and reception algorithm for the dual array method has been proposed. The time delay law of the dual array transducer for the complex turbine disk structure has been investigated. Finite-difference time-domain theory has been used to establish the numerical model of the dual array method. In the numerical simulations, the novel method has been applied for the superalloy turbine disk specimen with prefabricated defects at the depth of 18.3 m and 28.3 mm. Furthermore, the corresponding experiment has been conducted and verifies the reliability of the simulation. The novel method shows advantages in detecting small diffusion bonding defects in complex structure, assisting the manufacture of superalloy turbine disks, and ensuring the safety of aircrafts.

## Introduction

**Figure 1:**
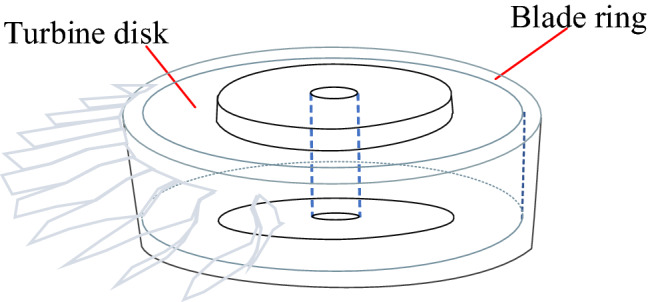
Turbine disk structure diagram.

Superalloy turbine disk is a key component of the aeroengine. As can be seen from Fig. 1, the main body of the turbine disk is connected with the blade ring by the diffusion bonding^1–3^. Due to the factors such as surface roughness and impurities during the welding process, diffusion defects are easy to occur on the welding interface, risking the robustness of the aero-engine. Unlike the volume defects such as holes and pores, these defects are planar defects, which are hard to be detected since the relative position of defects and ultrasonic transducers will greatly affect the detection results^4,5^. If the incidence direction of the sound beam is not perpendicular to the planar defect, the echo signal could be difficult to be received by the transmitting transducer. This is an important limit resulted from the turbine disk structure^6^.

**Figure 2:**
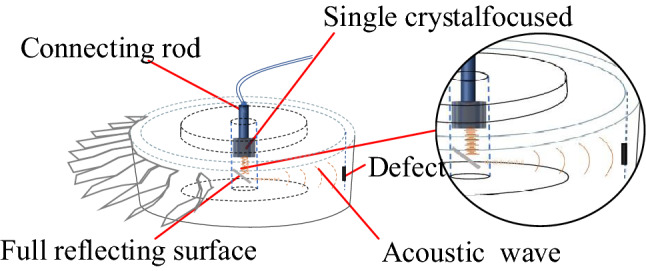
Schematic diagram of the main detection methods currently.

In addition, the complex structure of the tested superalloy turbine disk limits the space for the transducer arrangement. Currently, the commonly used detection method is the conventional monolithic transducer pulse reflection method. As shown in Fig. 2, in this method, the acoustic waves would be propagated in the disk. Thus, the signal-to-noise ratio of the defect echo is low and the detection resolution is poor.

Compared to standard monolithic transducers, ultrasonic arrays beam is flexible and controllable. It can conduct multi-angle and multi-direction scanning for the inspection objects. This can effectively reduce the defect missing rate and improve the detection reliability^7^. By firing the array elements in properly time-delayed pulses which are called the delay law, the parameters of sound beams such as focus depths and steering angles can be adjusted, consequently improving the accessibility to imaging defects^8^. Previous studies on the applications of ultrasonic phased array technique include detection for low-pressure turbine disk^9^, jet-engine turbine blades^10^, corner-shaped components^11^, and welds in blade specimen^12^. Yang et al.^9^ used phased array ultrasonic technique to inspect the stress corrosion cracking of the tenon teeth in the low-pressure turbine disk. This method improves the detection ability and resolution. Furthermore, Yang et al.^13^ used a combination of phased array ultrasonic testing and artificial neural network algorithms to locate and estimate the depth of the cracks in the low-pressure turbine disk. The General Electric Company^14^ adopted a ultrasonic microscope technology that can clearly show a flat-bottomed hole with a diameter of 0.254 mm and a buried depth of 3.18 mm on the Rene 95 board. Although these studies can detect small defects well, they are limited to inspect the surface defects, and their inspection efficiency is low. Additionally, although (time-of-flight -diffraction) ToFD^5^, (total focusing method) TFM^15^, ultrasonic phased array technique et, al all have been used in planar defects inspection, most of the detected objects are plate structures, which don’t belong to the complex structure. The non-destructive testing (NDT) method needs to be further improved to defect smaller defects in complex structure and enhance the safety and quality requirements of the superalloy disk.

This paper proposes a novel ultrasonic detection method using dual array ultrasonic transducers. A corresponding synthetic acoustic beam transmission-reception algorithm is designed for novel method. The applications of the novel method for a wide range of defect depths are simulated by numerical simulation. An experiment was conducted and verified the simulation results. It suggests that the novel dual array method can effectively improve the efficiency and accuracy of detection for the superalloy turbine disk and can be applied in different defect depth.

## Inspection scheme of the dual array method

### Ultrasonic beam transmission and reception algorithm for the dual array method

**Figure 3:**
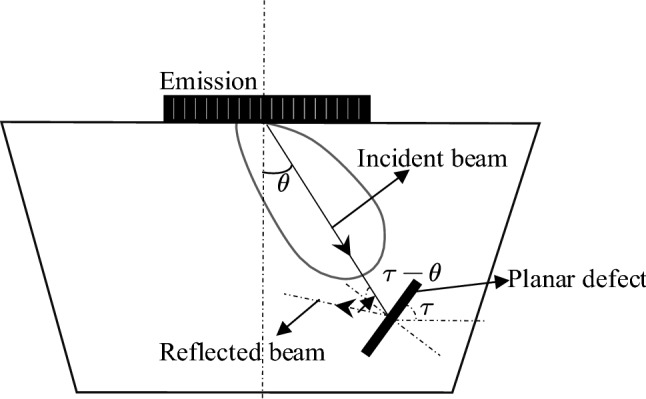
Schematic diagram of acoustic reflection of area defects deviating from the axis of the acoustic source.

The relative position of the defect and the ultrasonic beam source has a great influence on the detection result^16^. As shown in Fig. 3, assuming that the deflection angle of the central axis of the main sound beam is $\theta$, the inclination angle of the planar defect to the horizontal surface is $\tau$, and thus the angle between the incident wave and the defect plane is $\tau - \theta$. The acoustic pressure of the reflected wave at the center point of the transducer can be expressed as Eq. (1)^5^:

1 $$ P_{t} = \frac{{p_{0} r_{p} D_{L}^{2} \left( \theta \right)\sin \left[ {k_{L} {\text{csin}}\left( {\theta - \tau } \right)} \right]}}{{k_{L} {\text{ctan}}\left( {\theta - \tau } \right)}} $$

where $r_{p}$ is the acoustic reflection coefficient of the ultrasonic wave at the air-specimen interface, $D_{L} \left( \theta \right)$ is the directivity coefficient of the wave, $k_{L} = \frac{2\pi }{\lambda }$ is the wave vector, $\lambda$ is the acoustic wavelength. When ${\uptheta } \approx {\uptau }$, the reflected ultrasonic pressure of defects is at its maximum value. The axis of the main ultrasonic wave is perpendicular to the defect plane and the acoustic pressure of the defect echo received by the transducer is at its maximum value. When ${\uptau } - {\uptheta } > 0$, that is, when the inclination angle of the defect to the horizontal surface is larger than the angle between the central axis and the main beam, the transducer cannot receive the reflected ultrasonic wave after the main ultrasonic wave interacts with the defect. In this case, the tip diffraction signal of the planar defect is too weak to be used for defect inspection. Therefore, comprehensive consideration to the specimen structure and the ultrasonic wave transmission and reception method is important in designing an ultrasonic testing algorithm.

**Figure 4:**
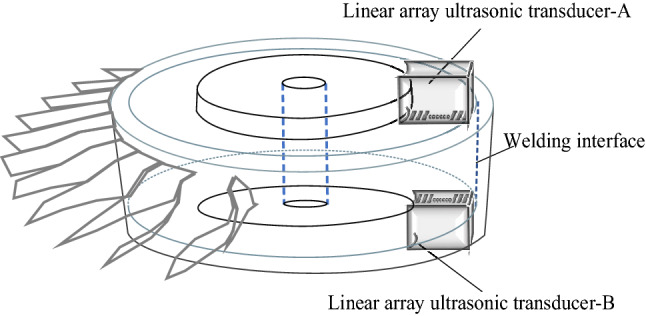
Schematic diagram of bilinear array transducer detecting diffusion weld of superalloy turbine disk.

Figure 4 shows the integral structure of the superalloy turbine disk and the layout of the dual array ultrasonic transducers. Since the defect plane is parallel to the welded interface, when the array ultrasonic transducer-A is transmitted, the defect reflection signal can be effectively received by the array ultrasonic transducer-B. However, due to the short horizontal distance between the array ultrasonic transducer and the inspection area, and the vertical distribution of the welds, according to Eq. (1), the reflected acoustic pressure of the defect is weak. If the acoustic beam deflection is used directly for detection, the defect signal will be very weak, even submerged by other signals. In addition, since the vertical welds of the superalloy turbine disk distribute on the entire specimen, the deflection and focus parameters of the acoustic beam need to be specifically designed to achieve the detection of the entire weld area. Therefore, in this algorithm, all the welds on the cylindrical surface of the superalloy turbine disk will be regarded as the inspection object. To achieve the full coverage inspection of the welding interface, the acoustic beam control scheme and imaging method based on dual array ultrasonic transducers is designed.

**Figure 5:**
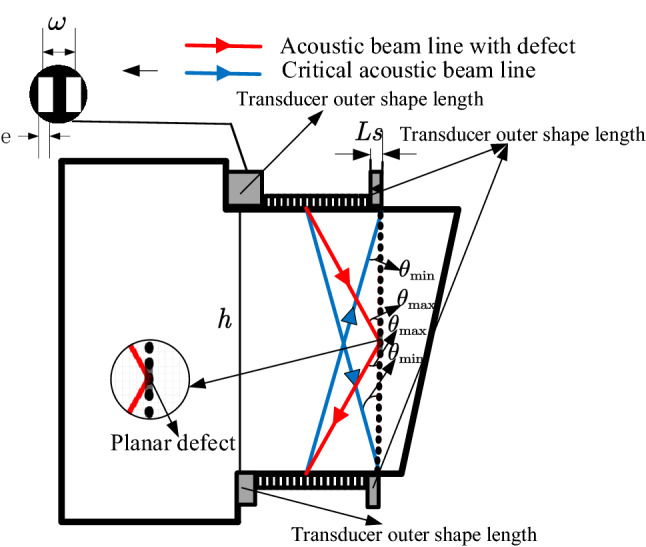
The dual array method ultrasonic testing scheme for superalloy disc.

The interaction between the array ultrasonic transducers and the large deflection angle of the sound beam have a great influence on the detection result^15^. To avoid this, first, the specific focus delay law is used for transmission of the array ultrasonic transducer-A and the reception of the array ultrasonic transducer-B. In this way, the lower part of the welds near the transducer-B can be detected. Then the transmission and reception transducer to detect the upper part of the welds, Fig. 5 shows the pitch-catch array ultrasonic testing scheme for superalloy disc.

Assuming that the quantity of array elements for each transducer is N/2, taking the array ultrasonic transducer-A as an example, the deflection angle ${\uptheta }$ of the synthesized acoustic beam is in $\left[ {\arctan \left( {\frac{{d \times \left( {N - 1} \right)/2 + e + L_{S} }}{h}} \right),\arctan \left( {\frac{{d \times \left( {N - 1} \right)/2 + e + L_{S} }}{h/2}} \right)} \right]$.

Where $d$ is the pitch of the array elements, *e* is the width of the array elements, $L_{s}$ is the transducer outer shape distance, this distance is also marked in Fig. 8, and *h* is the vertical height of the weld. Since the inclination angle of the planar defect to the horizontal surface, ${\uptau }$, is fixed at $90^\circ$, the angle of acoustic incidence waves to detect the defects at different locations can be calculated.

**Figure 6:**
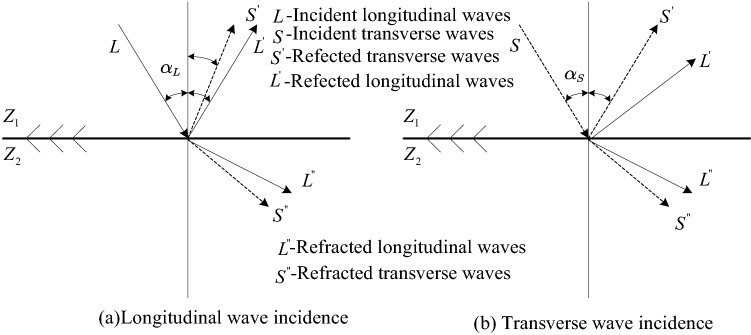
Mode conversion of reflected acoustic waves under different incident waveforms.

For different types of the incidence wave and the incidence angles, different waveform conversions will occur after being reflected by the interface and affect the evaluation of the defect signal. It is important to further confirm the type of focused acoustic wave. Figure 6 shows the waveform conversion with longitudinal waves and transverse waves at a certain incidence angle. Figure 6a shows the waveform conversion of the reflected sound wave with the longitudinal wave at an incident angle ${\mathrm{\alpha }}_{L}$. As the incident angle ${\mathrm{\alpha }}_{L}$ increases, the reflected ultrasonic wave will contain both longitudinal and transverse waves. Figure 6b shows that, for a transverse wave, as the incident angle ${\mathrm{\alpha }}_{s}$ increases, the longitudinal wave will disappear, and only reflected transverse waves will exist. Considering Eq. (2) and the geometric size of the superalloy disk, the minimum incidence angle of the ultrasonic wave in this detection scheme is $65.3^\circ$. If the longitudinal wave is used for focusing, most of the energy after the interacting with the defect will be converted into reflected transverse waves while reflected longitudinal waves exists at the same time, leading to a complex reflected signal and weak energy. Therefore, to ensure a simple reflected signal of pure transverse wave, the focused transverse wave is used for detection.

### Time delay law calculation

**Figure 7:**
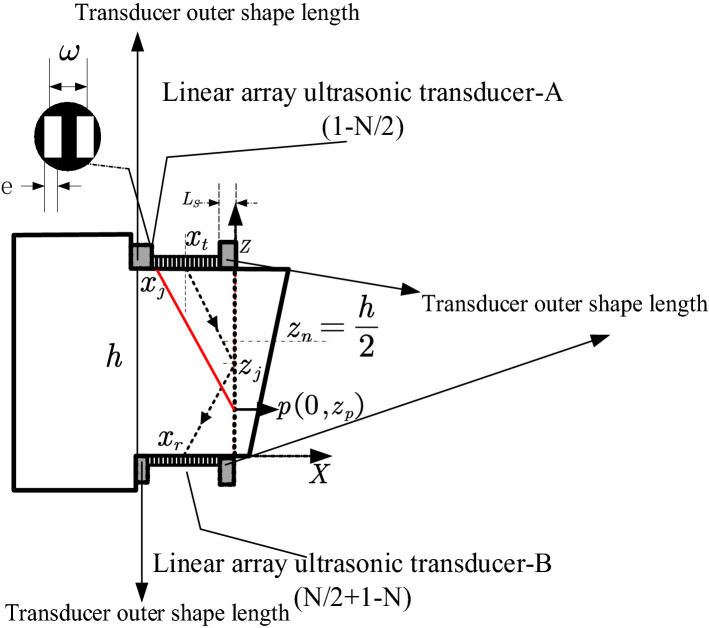
Delay time calculation of diffusion weld planar detection.

Different from the conventional array ultrasonic scanning method, for this specimen, the synthetic ultrasonic beam of the array ultrasonic transducer needs to change the focus depth of the current channel and the focus deflection angle. As shown in Fig. 7, taking the intersection point O of the weld and the bottom surface of the specimen as the origin of the coordination, it is assumed that the width of the array element is $$ w $$, array element pitch is $$e $$,the height of the weld is $$h$$, the transverse sound velocity in the specimen is $$c_{s}$$. When the ultrasonic beam is focused on the weld position $$p\left( {0,z_{p} } \right)$$, according to the Fermat’s principle, the wave propagation time of the element $$j$$ to the focal point $$p$$ is obtained by Eq. (2):

2 $$ T_{j} = \min \sqrt {\frac{{x_{j}^{2} + \left( {h - z_{p} } \right)^{2} }}{{c_{s}^{2} }}} \left( {z_{p} \le \frac{h}{2}} \right) $$

where

$$ x_{j} = - \frac{{2L_{S} + hd + w}}{2} + jd $$

$z_{p}$ is located on the Z-axis at a specific focus point. According to this, the value $T_{j}$ can be calculated. For a fixed focal point on the weld interface, Eq. (3) can be used to calculate the propagation time for the ultrasonic waves by all excitation array elements in the current channel. The maximum propagation time is defined as $T_{max}$ Therefore, the delay time ${\Delta }T_{j}$ corresponding to the element $j$ is calculated by Eq. (3):

3 $$ \vartriangle T_{j} = T_{max} - T_{j} $$

When the array ultrasonic transducer-A works for transmission, a certain number of focal points in the range of $Z_{0} - Z_{n}$ are discrete at the lower half of the weld near the array ultrasonic transducer-B. According to the time delay law, the delay time for the $\left( {n + 1} \right)$ focal point can be calculated. Similarly, when the array ultrasonic transducer-B works for transmission, according to the symmetry of the structure, the focus law of array ultrasonic-A can be directly applied to detect the upper half of the weld.

At the same time, the array ultrasonic transducer will also receive the sidewall reflection signals. Therefore, the total ultrasonic propagation paths of the defect signal from different positions of the weld to the reception transducer are different, that is, $\left| {x_{t} z_{j} } \right| + \left| {z_{j} x_{r} } \right|$ as shown in Fig. 7. Hence, after determining the discrete focus points, the total propagation time of the ultrasonic beam transmitting to each discrete point and then being reflected to the reception transducer need to be calculated. Correspondingly, for each channel, specific gate parameters need to be designed to accurately receive the reflected signals from different defects.

Assuming the superalloy turbine disk to be an isotropic medium, the acoustic velocity of the transverse wave remains unchanged as the propagation angle changes. If the abscissa of the center point of the transducer is $x_{t}$, when the synthetic ultrasonic beam of the array ultrasonic transducer-A propagates to the focal point $z_{i}$ and is reflected by the defect, the propagation time to the reception transducer-B is calculated by Eq. (4):

4 $$ t_{j} = \frac{{\sqrt {\left( {h - z_{j} } \right)^{2} + x_{t}^{2} } + \sqrt {z_{j}^{2} + \left( {\frac{{z_{i} x_{t} }}{{h - z_{j} }}} \right)^{2} } }}{{c_{s} }} $$

## Defect response simulations

### Transducer parameter selection

In array ultrasonic testing, the quality of the transducer has a great influence on the inspecting results. Only by selecting proper transducer parameters can the array ultrasonic transducer fire sound waves with good characteristics. The main parameters include element frequency, array element pitch, and elements number.

The center frequency $$ f$$ : the longitudinal acoustic velocity in the superalloy turbine disk is 5980 m/s. The resolution of the ultrasonic wavelength should be smaller than or equal to twice of defects size. To ensure the detection ability for defects with an equivalent diameter of 1 mm, the center frequency of the transducer should be above 5 MHz. Considering the manufacturing cost, the center frequency of the array transducer is selected to be 5 MHz.

Array element pitch $d$: array element pitch is the distance between the centers of two adjacent array elements. To ensure the acoustic beam directivity of the transducer, the element pitch of the array transducer should be half of the ultrasonic wavelength. At the center frequency of 5 MHz, the array element pitch $e$ should be 0.4 mm, and the array element width $w$ should be 0.3 mm.

Elements number *N*: generally, the more elements the array transducer has, the higher the acoustic pressure in the acoustic beam propagation direction and the signal-to-noise ratio would be. To improve the defect inspection resolution and detection accuracy, the number of array elements should be as large as possible. However, as the number of array elements increase, the number of channels to match array ultrasonic board will increase, resulting in higher cost.

**Figure 8:**
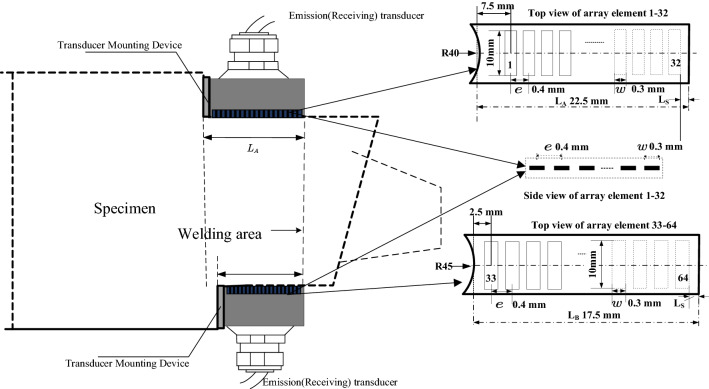
Schematic diagram of the placement of the transducer and the array element dimensions.

Moreover, the space available for the array ultrasonic transducer on the turbine disk is extremely narrow, limiting the number of transducer array elements. Assuming the distance from the upper rounded end of the turbine disk to the weld is $L_{A}$. As shown in Fig. 8, the number of array elements of the ultrasonic transducer is limited by:

$$ (N - 1)e + w < L_{A} $$

Here, the number of array elements for each transducer is selected to be 32. Since the dual array transducers has two array probes, the corresponding array ultrasonic board must have at least 64 channels.

**Figure 9:**
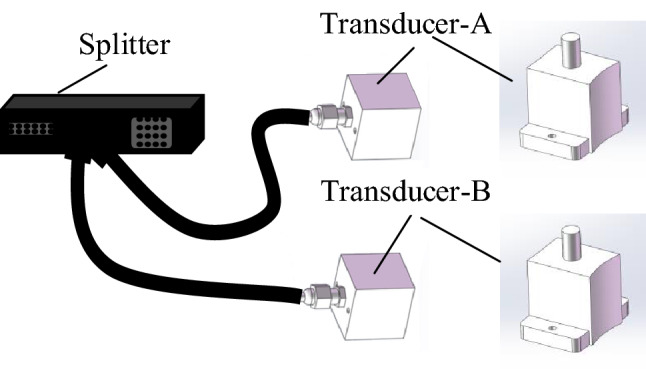
Transducer wiring diagram.

In summary, the parameter selection for the array ultrasonic transducer are listed in Table 1, and the schematic of the transducers is shown in Fig. 9.

**Table 1:** Array ultrasonic transducer inspecting parameters.

Parameter settings	Value
Center frequency	5 MHz
Elements number	32
Array element pitch	0.4 mm
Array element width	0.3 mm
Deflection angle	13.1$^\circ$–4.7$^\circ$
Focus depth	18.3–36.6 mm

### Selection of detection schemes

**Figure 10:**
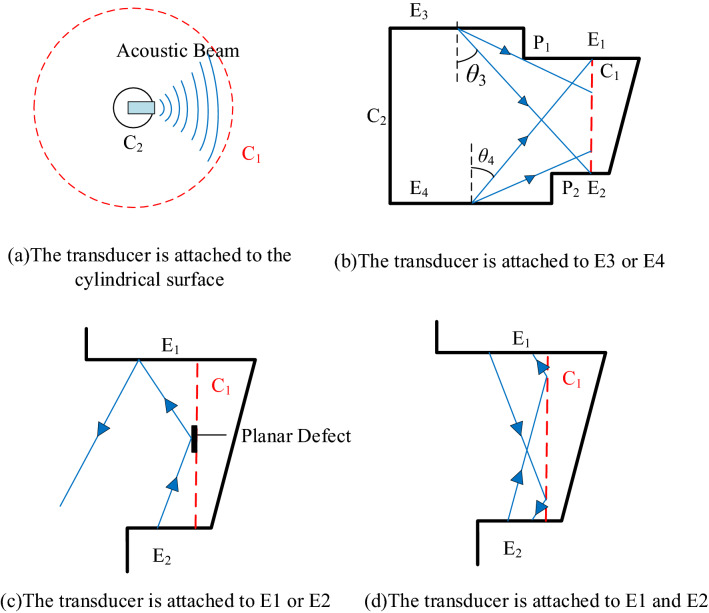
Difficulties in the detection of superalloy turbine disk.

Theoretically, to detect planar defects along the $Z$-axis, the acoustic wave transmitted by the transducer should propagate as far as possible in the direction perpendicular to the diffusion welding surface, the cylindrical surface $C_{2}$ as shown in Fig. 10a. However, as the acoustic wave propagates from $C_{2}$ to $C_{1}$, the focal point of the acoustic wave will gradually increase. In this case, only larger crack defects can be detected. The requirement of the minimum detection size cannot be satisfied.

When the transducer is on the edge E_3_ or E_4_, as shown in Fig. 10b, to make the acoustic wave propagate to $C_{1}$, the deflection angle of the synthesized acoustic beam should be greater than ${\uptheta }_{3}$(${\uptheta }_{4}$). However, according to the directivity formula, the energy of the acoustic beam propagating along an angle greater than ${\uptheta }_{3}$(${\uptheta }_{4}$)is relatively weak. This reduces the signal-to-noise ratio of the defect echo. In addition, the sharp point $P_{1}$($P_{2}$) is likely to interfere with the synthetic acoustic beam with a large deflection angle, resulting in the generation of artifacts. Therefore, when the transducer is on the edge $E_{1}$ or $E_{2}$, the acoustic beam can be transmitted to $C_{1}$ only with a small defection angle. It cannot meet the requirements of the welding surface detection and evaluation. When the acoustic wave propagates to the vicinity of the crack defect in the central area of the welding surface, most of the acoustic waves will be specularly reflected.

To ensure the transducer arranged on the side $E_{2}$ ($E_{1}$) to receive the acoustic wave energy by the specular reflection, as shown in Fig. 10c, the acoustic beam path should pass through the reflection of the side $E_{1}$ ($E_{2}$) at least once. When the number of reflection is 1, due to the limited length of the side $E_{2}$ ($E_{1}$), the geometric acoustic rays may propagate beyond the side $E_{2}$ ($E_{1}$) after being reflected by the side $E_{1}$ ($E_{2}$). This will make the transducer cannot receive the reflected defect signal. Since the specimen is thick, the number of reflections is greater than 1. The beam path will be fold increased, resulting in rapid decrease in defect echo signals and signal-to-noise ratio.

Finally, the dual array method is selected as can be shown in Fig. 10d, a dual array ultrasonic transducers are arranged on the side $E_{2}$ and $E_{1}$, respectively. For different detection positions in the weld area, the corresponding focusing rules are designed, by changing the transmitting transducer and the reception transducer to achieve the beam coverage of the whole welding region. To detect the weld area of the lower round end part, the array transducer below emits ultrasonic waves for deflection and focusing, and the ultrasonic transducer on the upper display receives and processes the signal. When the upper half of the weld area is to be detected, the lower array ultrasonic transducer emits sound waves for deflection and focusing, and the upper array ultrasonic transducer receives and processes the signal.

### Numerical simulation

**Figure 11:**
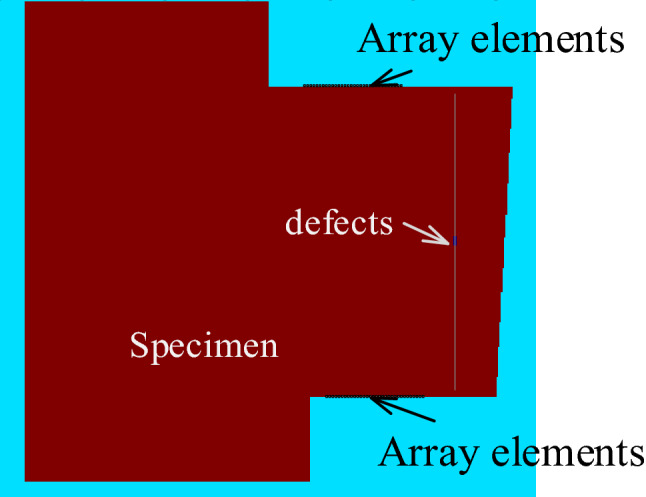
Array ultrasonic testing acoustic model of superalloy disc.

To analyze the applications of the novel dual array method, a two-dimensional simulation model for the detection of planar defects in superalloy turbine disk is established, as shown in Fig. 11. The grid size of the simulation model is 0.02 mm. The simulation interval time is set to be 1 ns, and the total time computation is 25 us. The pressure load is used as the array element excitation load, perpendicular to the ranking direction of the ultrasonic transducer array elements. Additionally, considering the smaller excitation energy of each array element, a 3-cycle, Gaussian window pulse with a center frequency of 5 MHz is used as the excitation signal for each array element. A rectangle planar defect is used for simulation, with a length of 1 mm and a width of 0.2 mm at the center of the weld at a depth of 18.3 mm and 28.3 mm. The longitudinal wave velocity in the superalloy turbine disk is 5980 m/s, and the transverse wave velocity is 3138 m/s.

**Figure 12:**
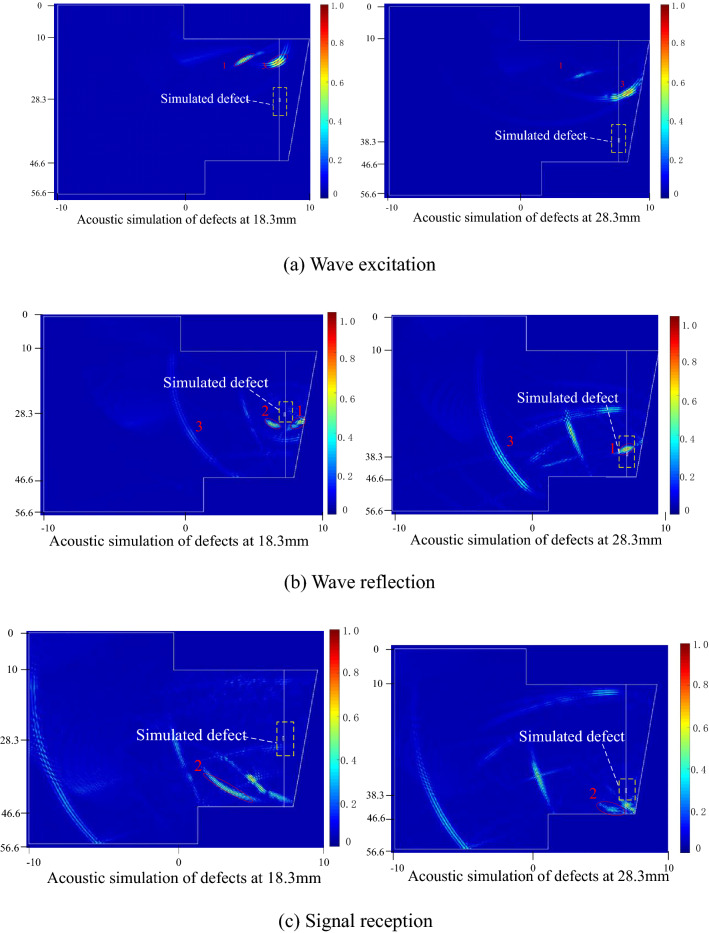
The wave propagation process and reflected the result of the focusing shear wave.

According to the proposed focused shear wave detection scheme using dual-array ultrasonic transducers, the propagation of the array ultrasonic synthetic beam and its effect on defects are shown in Fig. 12. At the initial moment shown in Fig. 12a, according to the focus law, the waves excited by the transmitting transducer-A include the focused transverse wave 1 and the longitudinal wave 3. When the focused transverse wave acts on the defect, part of the focused transverse wave 1 is incident on the defect surface obliquely to produce a reflected transverse wave 2, as shown in Fig. 12b. In Fig. 12c, the transverse wave 2 is reflected by the defect and the reflected signal from the sidewall will smoothly propagate to the array ultrasonic transducer-B attached to the other side of the superalloy disk.

**Figure 13:**
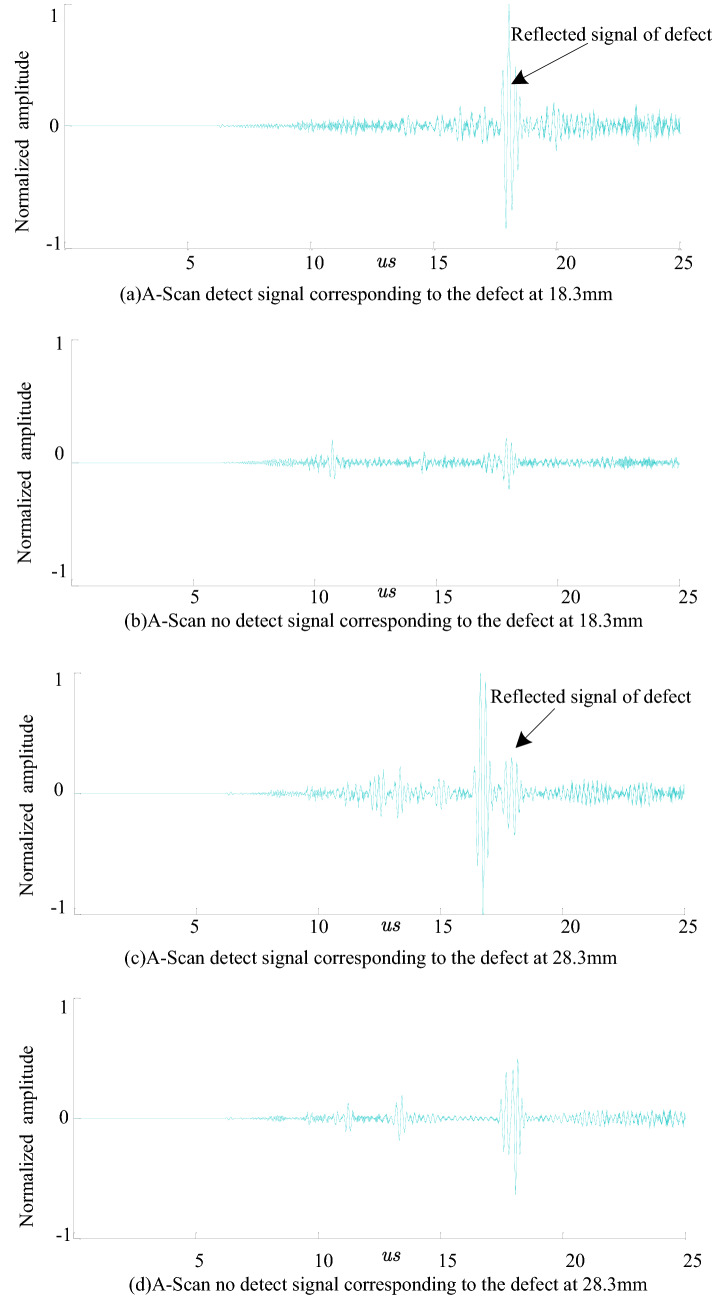
Simulation result of the planar defect detection with the dual array method.

The defect-free simulation model and the simulation model containing with planar defects are established. The signal received by each element is phase-delayed. The ultrasonic signal received by the transducer is extracted and drawn. The time from the defect signal corresponding to the channel to the reception transducer can be calculated according to Eq. (4). Figure 13 shows the A-scan signal received by the transducer B in the zero-defect model and the defect model at 18.3 mm and 28.3 mm, respectively. It can be seen that the proposed detection method has high detection signal-to-noise ratio and sensitivity to planar defects.

## Experiments

### Test rig

**Figure 14:**
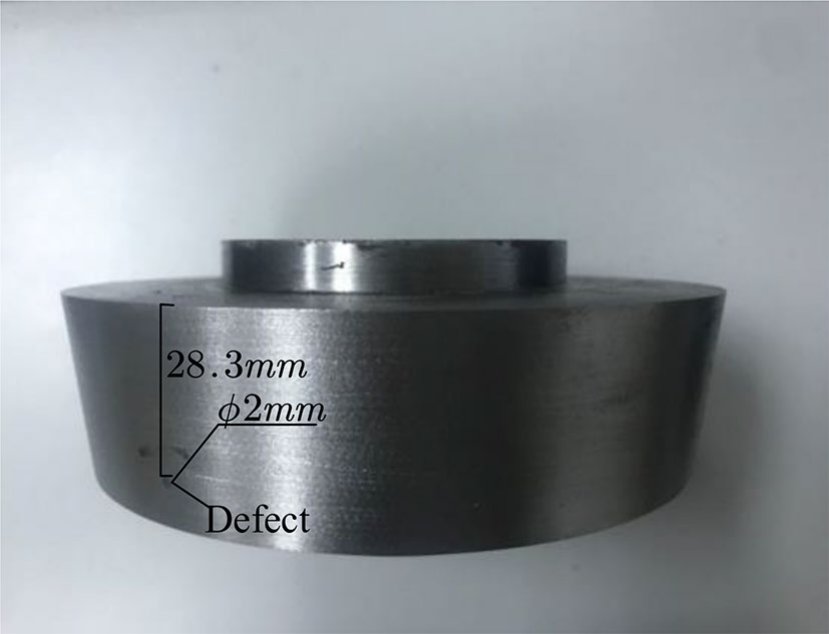
The superalloy disc sample.

The sample disk is made of carbon steel with an acoustic speed close to the superalloy. For the convenience of machining, the part of the disk body without blades is used as the test sample and the defects are prefabricated. A flat-bottomed hole with a diameter of 2 mm is machined at a depth of 28.3 mm from the upper surface on the outer wall of the specimen. The bottom of the hole is just at the welding interface to represent the planar defect in the weld. The specimen is shown in Fig. 14.

**Figure 15:**
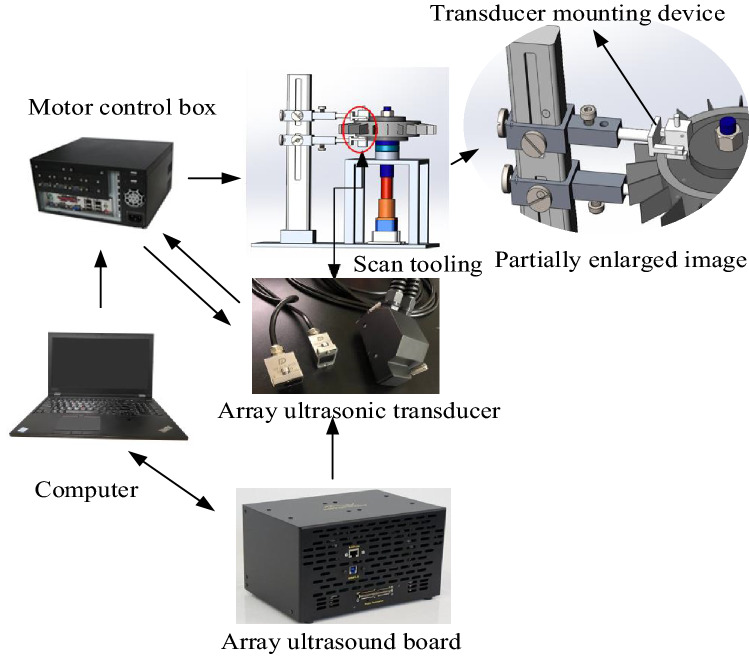
Automatic ultrasonic array testing system for superalloy disk.

According to the proposed inspection scheme, an inspection system using dual array ultrasonic transducers is established. The integral composition is shown in Fig. 15. Two array transducers are connected to the array ultrasonic board through a splitter. The scanning tool uses the bottom motor to drive the specimen to fix to the array ultrasonic transducer. The computer controls the array ultrasonic board and scanning tooling to realize automatic detection, and process the collected data to visualize the detection results of the entre diffusion welding interface.

**Figure 16:**
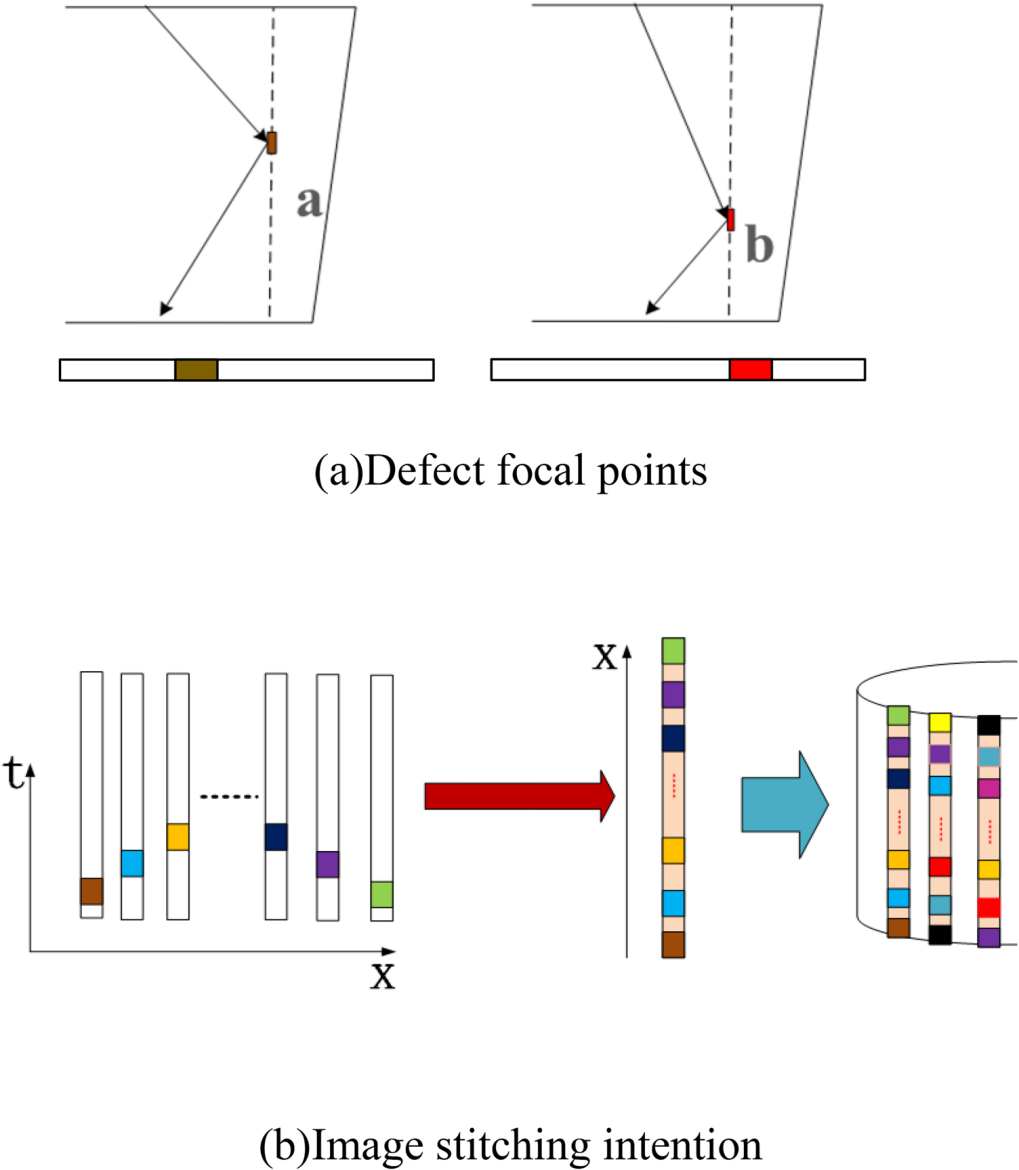
Image process diagram.

The discrete interval of the focus points along the depth direction of the weld is set to be 0.5 mm. Since the wave propagation path and time of each channel are determined, the gates corresponding to different channels can be used to extract the detect reflection signals at different positions of the weld. By changing the ultrasonic beam transmitting-receiving algorithm of the dual-array ultrasonic transducers, the entire weld can be inspected without moving the transducer. With the rotary scanning device, C-scan imaging of the entire disk can be realized. The schematic of the imaging process is shown in Fig. 16.

In addition, methods such as computer graphics processing unit parallel algorithms and Open GL 3D image mapping are used to realize automatic C-scan inspection and 3D real-time display of the overall weld planar defects of the disk specimen.

### Experimental results

**Figure 17:**
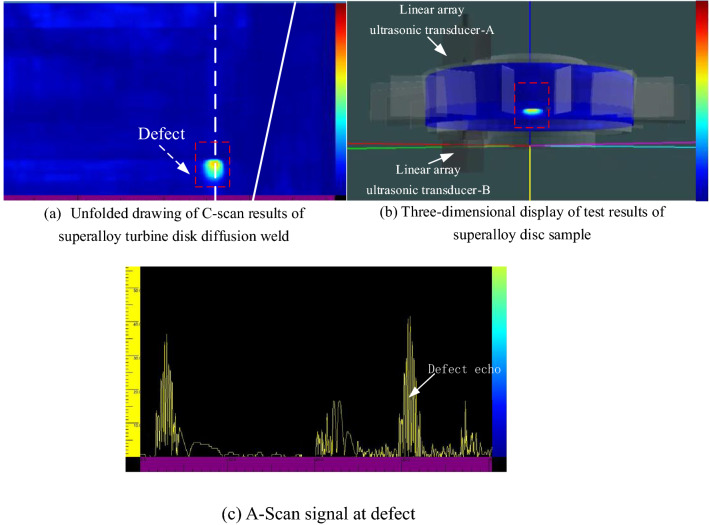
The detection results of the superalloy turbine disk sample using the dual array method.

The test result is shown in Fig. 17. The test results can clearly show the defect distribution in the weld area of the disk. Through comparison and verification, the position and size of the defects in the C-Scan test results have a good correspondence with the prefabricated defects in the specimen. This also verified the numerical simulation result that the reception transducer received most of the reflected echoes successfully. Compared with the conventional single crystal ultrasonic transducer inspection method, the novel dual array method has the advantages of the short beam path and flexible detection methods, and thus has high sensitivity to unwelded defects in cylindrical welds.

## Summary and conclusion

This work proposed a dual array method for the inspection of planar defects in the welding area on the cylindrical surface of the superalloy turbine disk. To facilitate the simulation of different detection schemes using the dual array method, a simulation method was established based on the finite-difference-time-domain method. The detection schemes of the dual array method were optimized by the simulation method. Besides, the ultrasonic inspection experiment of the superalloy turbine disk specimen with an artificial defect embodied in the welding interface was conducted, verifying the reliability of the dual transducer method. Finally, the following conclusions have been drawn.

1. A dual array method for the welding area of the cylindrical surface of the superalloy disk was established, the method has the advantages of high detection flexibility and wide acoustic beam coverage, which could be further extended to the detection of other complex structures and special components.
2. The finite-difference time-domain method could verify different transducer parameters, delay times, and focus waves, which could benefit to design and optimize detection schemes using multiple array ultrasonic transducers.
3. Combined with a mechanical scanning system, 2 mm equivalent defects could be defected, the test results show that compared with the single crystal ultrasonic transducer, the detection capability of the designed array inspection and imaging method is improved by more than 1.5 times, and the detection efficiency is improved by dozens of times.
4. Although the dual array method solved the inspection of the planar defects on the welding area of the superalloy turbine disk, there still exists very little detection blind spot in the upper and lower ends of the welding cylinder. The schemes optimization problem of the detection blind spot needs further research.

## Abbreviations

FDTD: Finite-difference time-domain
NDT: Nondestructive testing

## List of symbols

$r_{p}$: Acoustic reflection coefficient of the ultrasonic wave at the air-specimen interface
$D_{L} \left( {\uptheta } \right)$: The directivity coefficient of the wave
$k_{L}$: The wave vector (m)
$$d $$: The array element pitch (mm)
$$ N$$: The array element number
$e$: The array element width (mm)
$$h $$: The vertical height of the weld (mm)
$c_{s}$: The transverse sound velocity (m/s)
J: Array element index
$L$: The incident longitudinal wave
$S$: The incident transverse wave
$S^{\prime}$: The reflected transverse wave
$S^{\prime\prime}$: The refracted transverse wave
$L^{\prime}$: The reflected longitudinal wave
$L^{^{\prime\prime} }$: The refracted transverse wave
$$ p $$: Focal point
$z_{p}$: A specific focus point at Z-axis
$T_{j}$: The wave propagation time of the element $j$ to the focal point $p$
$T_{max}$: Maximum propagation time (μs)
$L_{S}$: The distance from transducer to the welding area (mm)
$p\left( {0,z_{p} } \right)$: A point $p$ on the weld
$x_{t}$: The abscissa of the center point of the transducer-A
$x_{r}$: The abscissa of the center point of the transducer-B
$$z_{j} $$: The jth discrete focus point of the weld
Γ_*T*_: The area where the surface of the array ultrasonic transducer closely adheres to the coupling medium and the specimen
*R*: Any point on the boundary ∂Ω or in Ω
*v*_*i*_(***r***, *t*): The velocity of the medium at point ***r***
$c_{L}$: The longitudinal acoustic velocity of the medium (m/s)
$f$: The center frequency (MHz)
$L_{A}$: The maximum length of the transducer can be placed (mm)
$C_{1}$: Large diameter outer cylindrical surface
$C_{2}$: Small diameter inner cylindrical surface
$E_{1} \sim E_{4}$: Upper and lower end faces, respectively
$$P_{1} \sim P_{2}$$: Sharp point

## Greek letters

$${\uplambda }$$: Acoustic wavelength (m)
${\uptau }$: The horizontal inclination angle of the planar defect (°)
${\uptheta }$: The deflection of the central axis of the main ultrasonic wave angle (°)
w: The array element width (mm)
${\upalpha }_{L}$: The longitudinal wave incident angle (°)
${\upalpha }_{S}$: The transverse wave incident angle (°)
$\partial \Omega$: The boundary of the simulation area
$$\Omega$$: Simulation area
*σ*_*i*_(***r***, *t*): The stress tensor of the medium at point ***r***
${\Delta }t$: Discrete intervals in time (ns)
${\Delta }h$: Discrete intervals in space
${{\Omega^{\prime}}}$: The sub-area of ${\Omega }$
${\Theta }_{3}$: The maximum angle at which the sound wave emitted from the end face of *E*_3_ can be deflected (°)
${\uptheta }_{4}$: The maximum angle at which the sound wave emitted from the end face of *E*_4_ can be deflected (°)
${\Theta }_{min}$: The minimum angle covered by the sound beam (°)
${\uptheta }_{max}$: The maximum angle covered by the sound beam (°)
